# The Emerging Role of Disturbed CoQ Metabolism in Nonalcoholic Fatty Liver Disease Development and Progression

**DOI:** 10.3390/nu7125501

**Published:** 2015-12-01

**Authors:** Kathleen M. Botham, Mariarosaria Napolitano, Elena Bravo

**Affiliations:** 1 Department of Comparative Biomedical Sciences, The Royal Veterinary College, Royal College St., London NW1-0TU, UK; kbotham@rvc.ac.uk; 2 Department of Haematology, Oncology and Molecular Medicine, Istituto Superiore di Sanità, Rome 00161, Italy; mariarosaria.napolitano@iss.it

**Keywords:** non-alcoholic fatty liver disease, non-alcoholic steatohepatitis, Coenzyme Q, antioxidant, oxidative stress, inflammation, steatosis

## Abstract

Although non-alcoholic fatty liver disease (NAFLD), characterised by the accumulation of triacylglycerol in the liver, is the most common liver disorder, the causes of its development and progression to the more serious non-alcoholic steatohepatitis (NASH) remain incompletely understood. Oxidative stress has been implicated as a key factor in both these processes, and mitochondrial dysfunction and inflammation are also believed to play a part. Coenzyme Q (CoQ) is a powerful antioxidant found in all cell membranes which has an essential role in mitochondrial respiration and also has anti-inflammatory properties. NAFLD has been shown to be associated with disturbances in plasma and liver CoQ concentrations, but the relationship between these changes and disease development and progression is not yet clear. Dietary supplementation with CoQ has been found to be hepatoprotective and to reduce oxidative stress and inflammation as well as improving mitochondrial dysfunction, suggesting that it may be beneficial in NAFLD. However, studies using animal models or patients with NAFLD have given inconclusive results. Overall, evidence is now emerging to indicate that disturbances in CoQ metabolism are involved in NAFLD development and progression to NASH, and this highlights the need for further studies with human subjects to fully clarify its role.

## 1. Introduction

Non-alcoholic fatty liver disease (NAFLD), a condition in which triacylglycerol (TAG) accumulates in the liver, is the most common liver disorder worldwide [1,2]. Its occurrence in the general population has been estimated to be 20%–30%, but in increasingly prevalent metabolic disorders, including obesity, diabetes type 2 and the metabolic syndrome, this rises to 70%–90%. NAFLD itself is considered to be relatively benign, but in some cases its progression leads to liver conditions such as non-alcoholic steatohepatitis (NASH), fibrosis, cirrhosis and carcinoma which are more serious and may be life-threatening [1,2]. A better understanding of the causes of the development and progression of the disease, therefore, is now of great importance from a public health perspective [1,2].

NAFLD is known to be a multifactorial disease, but the reasons why progression occurs in some cases but not others are not yet well defined [3,4]. Recent work, however, has indicated that oxidative stress resulting from both insulin resistance and hepatic steatosis is likely to be a major causal factor in the development of NAFLD, and may also play an important part in its progression to more serious conditions such as NASH [4,5,6,7]. Coenzyme Q (CoQ) (also called ubiquinone) is an important cellular antioxidant found in all membranes [8,9,10]. Supplementation of the diet with CoQ has been found to influence plasma antioxidant and inflammatory markers in healthy men [11] and in patients with coronary artery disease [12,13], and animal studies have demonstrated effects on liver oxidative stress and lipid metabolism [14,15,16]. Furthermore, there is clear evidence to indicate that NAFLD is associated with perturbation of plasma and hepatic CoQ levels [17,18,19], and recent studies have demonstrated that CoQ metabolism is altered in an animal model of the disease [20]. Thus, there is growing evidence to suggest that disturbances in CoQ metabolism may play a part in the development and progression of NAFLD.

## 2. CoQ Function and Metabolism

**Figure 1:**
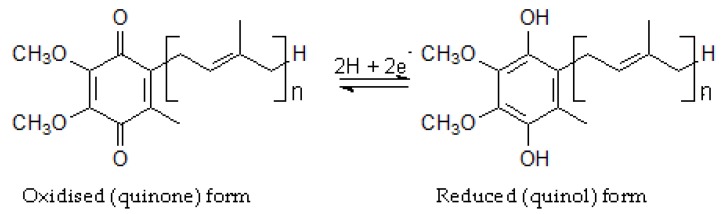
The oxidised and reduced forms of Coenzyme Q (CoQ). The oxidised and reduced forms of CoQ are interconverted by the addition or loss of 2H + 2e^−^. *n* = 10 in humans and 9 in rats and mice.

CoQ is a lipophilic molecule consisting of a benzoquinone ring and a polyisoprenoid side chain (Figure 1). The side chain varies in length from species to species, and for the purposes of this review it is important to note that in humans it contains 10 isoprene units (CoQ_10_), while in rats and mice, the most commonly used animal models for NAFLD, it has 9 (CoQ_9_) [21]. It is found in all cell membranes and is unique among naturally occurring lipophilic antioxidant in that it is synthesised *de novo* in animals [8,9,10]. The best known function of CoQ is in mitochondrial respiration. The side chain serves to keep the molecule anchored in the inner mitochondrial membrane, and the quinone ring, which is easily and reversibly reduced to the quinol form (Figure 1), enables it to carry out its function of transferring electrons from complexes I and II to complex III in the respiratory chain, ultimately resulting in the reduction of oxygen to water and the generation of ATP [10,21]. In addition to this essential role in energy generation, CoQ is now thought to have a number of other functions in the body, including regulation of mitochondrial functions such as membrane transition pore permeability and the activation of uncoupling proteins, counteraction of endothelial dysfunction by stimulating nitric oxide production, and, of particular interest in relation to NAFLD, acting as an anti-inflammatory agent and a lipophilic antioxidant protecting lipids, protein and DNA from oxidation [8,22,23].

CoQ is synthesised by a complex pathway involving the conjugation of the polyprenyl side chain with para hydroxybenzoic acid (PABA) the precursor of the benzoquinone moiety, followed by a series of modifications to the ring which result in the final structure [21,22,23]. At least nine enzymes are believed to participate in the pathway, and the steps specific to CoQ take place inside mitochondria. Isoprene monomers are synthesised via the mevalonate pathway in the cytosol, and are then condensed by the complex Pdss1/Pdss2, thus this enzyme determines the number of isoprene units in the CoQ side chain [21,22,23]. Deficiency of CoQ is known to occur in humans and leads to various mitochondrial disorders which have diverse clinical manifestations, including encephalomyopathy, ataxia, myopathy and a nephrotic syndrome [21,24,25]. The disorders may be primary or secondary. Primary disorders caused by defects in the biosynthetic pathway are rare, but mutations in a number of the genes in the pathway, including Pdss1 and Pdss2, have been identified (see reference [21] for a comprehensive list). Secondary deficiencies may result from defects in proteins unrelated to CoQ biosynthesis such as aprataxin, which has a role in double stranded DNA repair, mitochondrial DNA depletion, or the use of statins to combat hypercholesterolemia [21,24,25]. Although endogenous synthesis is normally able to meet the body’s need for CoQ, there are circumstances in which deficiencies not caused by genetic defects may occur. Statins, drugs widely used to lower blood cholesterol, can depress CoQ synthesis as they inhibit 3-hydroxy-3-methylglutaryl Coenzyme A reductase (HMG-CoA reductase), the rate-limiting enzyme in the pathway of cholesterol synthesis which includes the formation of the isoprenoid units required to produce CoQ [21,26]. In addition, CoQ levels may be pathologically changed in conditions associated with oxidative stress and in aging [27]. Current evidence suggest that dietary CoQ supplementation may be beneficial in many of these cases, but it is not yet used routinely in clinical practice [24,28].

### Antioxidant and Anti-Inflammatory Functions of CoQ

CoQ is biosynthesised in all tissues of the body, and, although it was initially believed to be confined to the inner mitochondrial membrane because of its function in the respiratory chain [29], it is now know to be present in all cell membranes [8,23]. Because of its widespread distribution and membrane location, CoQ is the most highly efficient of the naturally occurring lipophilic antioxidants [8]. In the membrane CoQ is in close proximity to lipid and protein molecules in need of protection from locally produced peroxy radicals. Much of the CoQ found in the body is in the antioxidant quinol form (Figure 1), although this varies between tissues [8]. The quinol prevents lipid peroxidation by inhibiting the initial formation and propagation of lipid peroxy radicals (LOO), and in the process it is oxidised to the quinone (Figure 1) and H_2_O_2_ is produced. In addition, it has been shown to protect proteins from oxidation by a similar mechanism [30], and to prevent oxidative DNA damage such as strand breakages [8,31]. As well as its role in membranes, CoQ is also believed to function in the blood to protect lipoproteins such as very low density (VLDL), low density (LDL) and high density (HDL) lipoproteins from oxidation [8]. The oxidised quinone product is rapidly and efficiently reduced to the quinol by enzyme systems in both the blood and the tissues, thus maintaining a high level of the active form [8].

**Figure 2:**
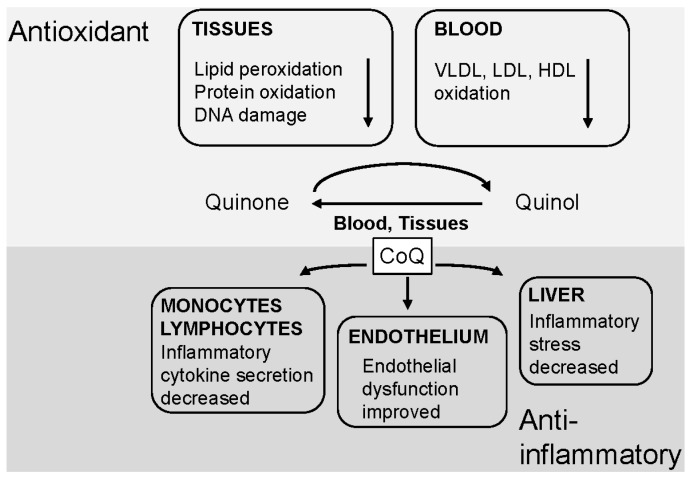
Antioxidant and anti-inflammatory functions of CoQ. Lipids, proteins and DNA and lipoproteins in the blood are protected from oxidative damage by the conversion of the quinone form of CoQ to the quinol form. The quinol is rapidly reconverted to the quinone in both blood and tissues. CoQ has also been shown to decrease cytokine secretion in monocytes and lymphocytes, to improve endothelial function and to reduce hepatic inflammatory stress.

As well as acting as an efficient antioxidant, current evidence suggests that CoQ has a number of independent anti-inflammatory effects [31,32]. It has been shown to reduce the secretion of pro-inflammatory cytokines in monocytes and lymphocytes after an inflammatory stimulus via an influence on the expression of NF-κB-dependent genes [14,24,33,34]. Furthermore, dietary supplementation with CoQ_10_ has been reported to improve endothelial dysfunction in patients with diabetes by up-regulating nitric oxide production (patients received 200 mg CoQ_10_/day for 12 weeks) [24,35,36], and to decrease the hepatic inflammatory stress caused by obesity in mice (see below) [14].

CoQ therapy has an obvious application in mitochondrial disease [28,37], but its antioxidant and anti-inflammatory effects have led to considerable interest in its possible beneficial effects in other chronic diseases, in particular cardiovascular disease [38,39], but also in neurodegenerative disorders including Parkinson’s disease, Huntington’s disease and Freidreich’s ataxia [38,40,41,42]. It has also been reported to reduce the risk of pre-eclampsia in pregnancy and to increase sperm quality in infertile men [38,43,44]. The list of conditions influenced by CoQ continues to grow, and below we present evidence from recent studies that suggests that changes in its metabolism may also have a role in NAFLD development and progression. The antioxidant and anti-inflammatory functions of CoQ are summarised in Figure 2.

## 3. Pathogenesis of NAFLD Development and Progression

NAFLD is defined as the presence of abnormal fat deposition in the liver (steatosis) without inflammation in individuals who do not consume excessive amounts of alcohol, and its occurrence is strongly associated with metabolic disorders such as obesity, diabetes and the metabolic syndrome [1,2]. The simple steatosis may progress to non-alcoholic steatohepatitis (NASH) (about 25% of cases), in which the fat accumulation is accompanied by inflammation and hepatocyte ballooning. Approximately 20% of NASH patients will develop fibrosis and cirrhosis, and in 1% of cases per year cirrhosis progresses to liver carcinoma [2]. The hepatic diseases and main clinical conditions involved in NAFLD development are well known and described in terms diagnostic criteria [45], and, although the pathogenesis from healthy liver to the disease condition remains incompletely understood, oxidative stress and insulin resistance have been strongly implicated as causative factors [5,46].

The finding that the risk of progression of NAFLD to NASH is related to the severity of fat deposition led Day *et al.* [47] to propose the “two hit” hypothesis in 1998. The first hit is the simple hepatic steatosis which results from the development of insulin resistance, and this leads to conditions in which the liver becomes more sensitive to the second hits which include oxidative stress, mitochondrial dysfunction and inflammation [2,4,47]. More recently, however, it has become clear that polymorphism in the gene for patatin-like phospholipase 3 (PNPLA3) also plays a role in NASH development, thus it is more accurately described as a multiple hit process [4].

**Figure 3:**
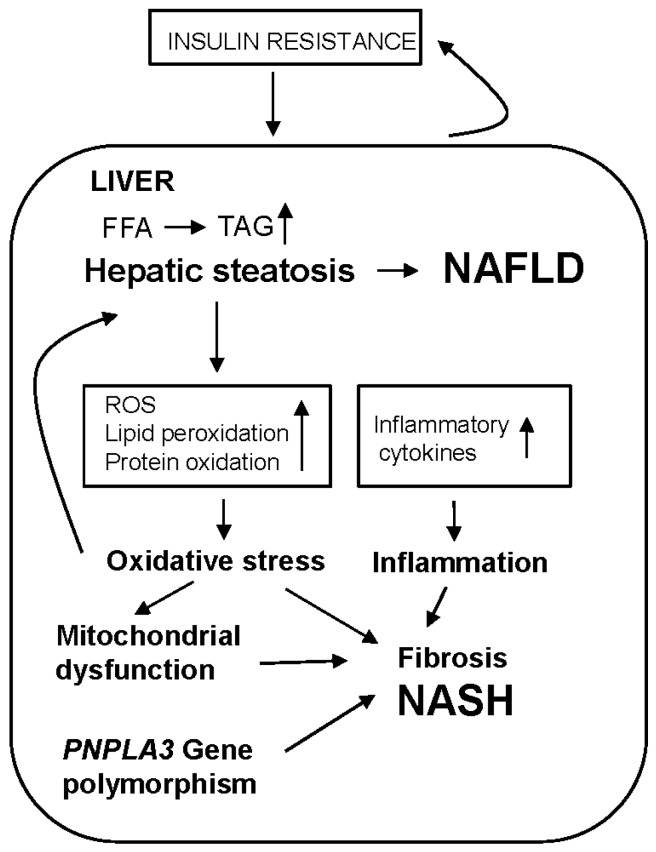
The development and progression of NAFLD. The development of insulin resistance increases the supply of free fatty acids (FFA) to the liver causing increased TAG synthesis and hepatic steatosis. Oxidative stress may also contribute to this process. The steatosis leads to inflammation and increased oxidative stress, which in turn causes mitochondrial dysfunction. These conditions, together with other factors such as *PNPLA3* gene polymorphism, result in the development of fibrosis and ultimately NASH. In the traditional 2 hit hypothesis [48] the initial steatosis is the first hit and the second hits are the resulting oxidative stress and inflammation. It is now clear, however, that multiple hits are involved in the process.

In the normal liver, there is a balance between the synthesis and esterification of fatty acids (FFA) which increases hepatic TAG content, and VLDL secretion and fatty acid oxidation which decreases it [48]. In insulin resistance, however, there is an increased supply of fatty acids to hepatocytes, causing not only fat accumulation but also a rise in mitochondrial β-oxidation, and consequently increased oxidative stress caused by raised levels of reactive oxygen species (ROS), lipid peroxidation, protein oxidation and the production of pro-inflammatory cytokines [5,49]. Recent studies have also suggested that oxidative stress may be a primary cause of liver fat accumulation [50], and ROS have also been shown to play a part in fibrosis development [51]. In addition, blood antioxidant concentrations have been reported to be decreased in patients with NAFLD [52]. It is now clear, therefore, that oxidative stress is a major factor in the development of NAFLD and its progression to NASH. Furthermore, a large proportion of cellular ROS are produced in mitochondria, and overproduction of ROS when mitochondrial respiration is disrupted has been suggested to be important in NAFLD development [53,54,55]. The presence of mitochondrial dysfunction in patients with NASH also indicates that it is likely to have important role in the pathogenesis of the disease [56,57]. Figure 3 summarises current ideas on the pathogenesis of NAFLD development and progression.

## 4. CoQ and NAFLD Development and Progression

Both the role of CoQ in mitochondria and its function as an efficient endogenously synthesised antioxidant [8,10,21] suggest that it may play a part in the development of NAFLD and its progression to NASH, and evidence for this has begun to emerge from recent investigations. In general, two main approaches have been used. The first has been to study the relationship between the disease and CoQ metabolism in the body and the second to assess the influence of dietary supplementation with CoQ on the development of the condition. So far, the majority of studies have used animal models of NAFLD or NASH, mainly in rats and mice, but as interest grows, more human studies are being undertaken.

Studies to date on how CoQ metabolism is changed in NAFLD have given conflicting results. As early as 1996, Eaton *et al.* [18] suggested from their work on β-oxidation in patients with alcoholic and non-alcoholic liver disease that decreased amounts of CoQ may contribute to hepatic steatosis in NASH , and more recently, Yesilova *et al.* [19] found that CoQ_10_ levels were depleted in men with NAFLD in comparison with normal subjects. Moreover, these authors also showed that there was a negative correlation between CoQ_10_ and body fat and also inflammatory activity and fibrosis [19]. In an investigation with obese rats, exposure of the animals to a mixture of environmental contaminants implicated in NAFLD development and progression has been reported to cause a decrease in liver CoQ_10_ concentrations [57], and the activity of geranylgeranyl pyrophosphate synthase, an enzyme involved in the formation of isoprene units, was also decreased in these conditions. The authors conclude from this that *de novo* synthesis of CoQ is depressed in NAFLD, but measurement of levels of CoQ_9_, the main endogenous form in rats, would be required to confirm this. In contrast, Bravo *et al.* [20] found that plasma levels of CoQ_9_ were increased in rats with high fat diet-induced NAFLD, while concentrations of CoQ_10_, which is likely to be mainly derived from exogenous sources, were not significantly changed. Furthermore, there was an increase of about 75% in the proportion of total plasma CoQ (CoQ_9_ + CoQ_10_) in the reduced form. Another study using a mouse model of NASH found increased levels of plasma CoQ_9_ in the animals, and the rise was positively correlated with hepatic ROS concentrations, hypercholesterolemia and fibrosis [58]. Since increases were observed in CoQ_9_, but not CoQ_10_ in these experiments [20,58], it seems likely that this change reflects up-regulation of endogenous synthesis which may be an protective response against a general reduction in the antioxidant capacity of the plasma which occurs in NAFLD [15,52]. In addition, respiratory activity and the activity of liver mitochondrial NADH-CoQ oxidoreductase (complex I of the respiratory chain) have been shown to be decreased in rats with NAFLD induced by choline deficiency [9]. Current evidence, therefore, clearly indicates that CoQ metabolism is disturbed in NAFLD, but further studies are required to elucidate how exactly this relates to the development and progression of the disease.

The finding that changes in CoQ metabolism are potentially involved in the development and progression of NAFLD raises the question of whether dietary supplementation with the quinone may be beneficial in the prevention or treatment of the condition. Although only a few studies have investigated the direct effects of CoQ supplementation on NAFLD (see below), a number of investigations in both humans and animals have provided evidence in areas known to be associated with the disease such as oxidative stress and mitochondrial dysfunction (studies on dietary supplementation with CoQ are summarised in Table 1). Despite the fact that CoQ_9_ is the endogenous form in rats and mice, with just a few exceptions (see below), investigations with animals have used CoQ_10_ as the dietary supplement.

Dietary supplementation with CoQ_10_ (300 mg/day for 12 days) has been found to reduce serum markers of oxidative stress in young swimmers [59] and, in a series of studies in patients with coronary artery disease (60–300 mg CoQ _10_/day for 12 weeks) , to reduce oxidative stress and increase the activity of antioxidant enzymes as well as lowering inflammation as assessed by plasma levels of inflammatory markers such as tumour necrosis factor (TNF)-α and interleukin (IL)-6 [12,13,60,61]. Similarly, dietary CoQ (diet supplemented with 0.07%–0.7% (w/w) CoQ_10_ for 26 weeks) was associated with a reduction in plasma oxidative stress and inflammatory markers in a rat model of the metabolic syndrome [62]. In healthy sedentary men given oral CoQ_10_ (100 mg/day for 8 weeks), however, Gobkel *et al.* [11] reported that blood levels of TNF-α and interleukin (IL)-6 were unchanged over the 8 week experimental period.

In the liver in general, animal studies have shown that dietary CoQ_10_ is hepatoprotective against toxic agents including acetaminophen (rats were given a single oral dose of acetaminophen (700 mg/kg), two injections (i.p.) of CoQ_10_ at 1 and 12 h and killed at 24 h) and carbon tetrachloride (a single i.p. dose of 200 mg/kg CoQ_10_ was administered 24 h before injection of CCl4 (1 mL/kg) s.c. and animals were killed 24 h later) in rats [63,64], inhibits liver fibrosis induced by dimethylnitrosamine (DMN) (10 or 30 mg CoQ_10_ given orally before administration of mDMN and 3 times/week thereafter for 3 weeks) or eggs of the parasitic worm *Schistoma mansoni* (*S. mansoni*) (oral dose of 5 mg/kg CoQ_10_ daily for 8 or 12 weeks after infection) in mice [65,66], and limits ischemic damage in transplanted livers when the donor rat is pretreated (10 mg/kg CoQ_10_ given i.v. 1 h before induction of ischemia) [67]. Intraperitoneal administration of CoQ_10_ (8 mg i.p. every second day for 38 days) has been shown to lower liver TAG content in rats [68], suggesting that it could be protective against hepatic fat deposition in NAFLD. Hepatic oxidative stress and inflammation has also been found to be reduced by CoQ_10_ therapy in several animal studies. In a model of diet-induced obesity, a high risk condition for NAFLD development in mice, Sohet *et al.* [14] showed that the expression of mRNA for a panel of hepatic genes involved in ROS production, inflammation and metabolic stress was reduced in the animals fed CoQ (diet supplemented with 1% CoQ_10_ for 60 days), and Othman *et al.* [66], also found that the quinone decreased oxidative stress and preserved antioxidant factors in livers of mice infected with *S. mansoni* (study details given above). In contrast, in another study although both CoQ_9_ and CoQ_10_ levels in the liver were increased in rats fed a diet supplemented with CoQ_10_ (62 mg/kg) for 6 or 12 months, the activities of the hepatic antioxidant enzymes measured were either unchanged or decreased [69]. Hepatic mitochondrial dysfunction has also been shown to be improved by CoQ_10_ (0.57 mg/day for 6 weeks) in rats given a high cholesterol diet and atorvastatin [70] and in cultured human hepatocytes treated with d-galactosamine, an inducer of oxidative stress and cell death and 30 μM CoQ [71].

**Table 1:** Summary of studies on the effects of dietary CoQ supplementation on NAFLD and related factors.

Subjects/Animals/Cells	Oxidative Stress (OS)	Inflammation	Other	References
Young swimmers	Plasma OS markers MDA, NO, protein hydroperoxide decreased; total antioxidant capacity increased			[59]
Patients with coronary artery disease	Plasma MDA reduced; SOD, CAT, GPx activites increased	Plasma TNF-α, IL-6 reduced		[12,13,60,61]
Rats with the metabolic syndrome	Plasma oxidised LDL decreased	Plasma hsCRP reduced	Endothelial dysfunction improved	[62]
Healthy sedentary men		Plasma TNF-α, IL-6 levels unchanged		[11]
Rats with liver injury induced by acetominophen or CCl_4_	Liver GSH increased; lipid peroxides decreased		Liver tissue damage ameliorated; liver NF-κB, caspase 3 and inducible NO synthase	[63,64]
Mice with diet-induced obesity	Liver expression of NADPH oxidase decreased	Liver expression of CRP, STAMP2 decreased	Liver expression of CPT1α decreased	[14]
Mice with liver fibrosis induced by DMN or *S mansoni*	Liver MDA decreased; GSH increased	Liver TGF-β reduced, Nrf2/ARE activated	Liver fibrosis decreased	[65,66]
Liver transplantation donor rats			Ischemic damage in transplanted liver prevented	[67]
Rats (CoQ_10_ given IP)			Lipid content of liver decreased	[68]
Rats fed sunflower oil or olive oil	Liver antioxidant enzyme activities unchanged or decreased			[69]
Rats fed a high cholesterol diet and atorvastatin			Serum and liver cholesterol and TG lowered; mitochondrial respiration improved	[70]
Rats with diet-induced NAFLD *	Plasma oxidised CoQ_9_ increased		Liver injury, steatosis, VLDL production unchanged; microsomal apoB, TG and membrane phospholipid increased; plasma leptin increased	[15,16]
Cultured human hepatocytes treated with d-galactosamine	ROS generation decreased		Electron transport chain dysfunction improved	[71]
Humans with NAFLD	Serum total antioxidant capacity decreased		Serum AST decreased, waist circumference decreased	[46]
Humans with NAFLD	Oxidative stress staus unchanged		No beneficial effects on serum lipid profile or blood pressure	[72]

ARE: antioxidant response element; AST: aspartate aminotransferase; CAT: catalase; CPT1α: carnitine palmitoyl transferase 1α; CRP: *C*-reactive protein; DMN: dimethylnitrosamine; GPx: glutathione peroxidase; GSH: reduced glutathione; hsCRP: high sensitivity CRP; MDA: malondialdehyde; SOD: superoxide dismutase; STAMP2: six transmembrane protein of prostate 2. *CoQ_9_ was used as the dieatry supplement. In all other studies listed CoQ_10_ was used.

The studies discussed above suggest that CoQ therapy may be beneficial effect in alleviating some of the causal factors of NAFLD, including oxidative stress, inflammation and mitochondrial dysfunction. Building on this base, our group investigated the direct effects of dietary supplementation with CoQ_9_ on the development of the disease using a high fat diet- induced model in rats [15,16]. CoQ_9_ was used rather that CoQ_10_ as it is the main endogenously synthesised form in the test species [21]. We used a dose of 30 mg/kg/day CoQ_9_ for 8 weeks after feeding a high fat diet for 10 weeks. NAFLD was found to be associated with increased, rather than reduced, plasma CoQ_9_ levels, while CoQ_10_ (mostly originating from the diet) concentrations were unaffected. However, the ratio of CoQ_9_:CoQ_10_ was significantly higher in NAFLD rats given the dietary supplement and plasma oxidized CoQ_9_ levels were also raised, effects which may be responses to increased oxidative stress [15]. Dietary CoQ_9_ was not found to have significant effects on hepatic steatosis, liver injury, the development of insulin resistance or increased production of VLDL seen in NAFLD, although it did cause a rise in plasma leptin levels, an effect which is likely to be detrimental rather than beneficial.

Under normal physiological conditions, the liver produces and secretes VLDL when energy for peripheral tissues is needed. TAG is transferred into the endoplasmic reticulum (ER), where it enters the VLDL precursor pathway [73]. In NAFLD, however, there is overproduction of large, TAG-rich VLDL causing hypertriglyceridemia [74]. Since a number of crucial steps in VLDL synthesis depend on the proper functioning of the ER membrane [73], Cano and colleagues carried out another study to investigate the effects of dietary CoQ_9_ on hepatic VLDL processing in rats with NAFLD [16]. CoQ_9_ was shown to have a number of effects including increasing microsomal levels of apoB and TAG and changing the microsomal membrane phospholipid content [16]. This kind of membrane change could delay the transfer of the nascent lipoproteins out of the ER into the secretory pathway, triggering increased apoB synthesis and TAG-rich VLDL particle size, and leading to the secretion of larger particles while the total concentration of VLDL TAG remains unchanged. Thus, although dietary CoQ_9_ was shown to affect VLDL secretion in hepatic steatosis, the changes may not be beneficial as they could lead to the production of larger, more atherogenic VLDL.

In human subjects with NAFLD, oral CoQ_10_ (100 mg/day for 4 weeks) has been reported to reduce waist circumference and decrease serum levels of aspartate aminotransferase, but was also found to decrease serum total antioxidant capacity [46], and in a second study (100 mg daily for 12 weeks), no beneficial effects were found [72]. Clearly, in considering dietary supplementation with CoQ as a therapy for NAFLD, it is important to establish whether it is able to bring about a reduction in hepatic fat accumulation and to prevent the development of fibrosis and carcinoma in the longer term. Because of the limited number of studies which have investigated effects on NAFLD directly, however, conclusive evidence is not yet available in these areas. CoQ administration has been reported to decrease hepatic steatosis in rats in one study [68], but in our work using a rat model of NAFLD we found no change [15]; liver fibrosis and hepatocellular carcinoma have been shown to be inhibited in rodents [65,66,75], but in these cases the conditions were induced chemically rather than as a result of NAFLD/NASH. Overall, therefore, studies to date, have not shown any direct benefit of CoQ therapy in combatting NAFLD development, however, as they are so few there is clearly a need for further investigations, and in particular for more human intervention studies, before any definitive conclusions can be reached.

## 5. Summary and Conclusions

Recent research has shown that oxidative stress, mitochondrial dysfunction and inflammation are key factors in the development of NAFLD and its progression to NASH [2,4,47]. The essential role of CoQ in mitochondria, therefore, together with its function as an efficient, endogenously synthesised antioxidant and its anti-inflammatory properties [8,10,21,24] suggest that disturbances in its metabolism may be involved in the pathogenesis of the disease, and evidence for this is now emerging. Investigations, mainly using animal models so far, have shown that CoQ metabolism is altered as NAFLD progresses, although its exact relationship to the disease process remains to be determined. Experiments with both animals and humans have demonstrated that dietary supplementation with CoQ is potentially beneficial for NAFLD, since it reduces oxidative stress and inflammation, improves mitochondrial dysfunction and is generally hepatoprotective. However, only a few studies either in animal models or in patients have assessed the direct effects of dietary CoQ in NAFLD to date, and the results have been inconclusive. Despite this, current evidence clearly indicates that CoQ is likely to be involved in NAFLD development and further studies, particularly with human subjects, are needed both to fully elucidate its role and to resolve the question of the efficacy of CoQ therapy in alleviating the effects of the condition.
